# Anti-Biofilm Properties of Cell-Free Supernatant from *Bacillus velezensis* EA73 by In Vitro Study with *Staphylococcus aureus*

**DOI:** 10.3390/microorganisms13051162

**Published:** 2025-05-20

**Authors:** Ziyao Tang, Muhammad Yousif, Samuel Kumi Okyere, Fei Liao, Siqi Peng, Lin Cheng, Feng Yang, Yuting Wang, Yanchun Hu

**Affiliations:** 1 Key Laboratory of Animal Disease and Human Health of Sichuan Province, Sichuan Agricultural University, Chengdu 611130, China; 2 Department of Veterinary Surgery & Obstetrics, Faculty of Veterinary Sciences, Shaheed Benazir Bhutto University of Veterinary and Animal Sciences (SBBUVAS), Sakrand 67210, Pakistan; 3 Department of Animal Husbandry and Fisheries, Guizhou Vocational College of Agriculture, Qingzhen 551400, China

**Keywords:** *Bacillus velezensis*, *Staphylococcus aureus*, biofilms, cell-free supernatant, anti-biofilm

## Abstract

Endophytes are a potential source of novel bioactive antimicrobial compounds. A previous study showed that *Bacillus velezensis* EA73, an endophytic bacterium, has promising antibacterial activity against *Staphylococcus aureus*; however, the mechanisms associated with its activity have still not been investigated. Therefore, this study was conducted to investigate the molecular mechanisms involved in the anti-biofilm activity of a cell-free supernatant (CFS) of *B. velezensis* EA73 against *S. aureus*. In this experiment, the biofilm-eliminating effects of a CFS of *B. velezensis* EA73 against *S. aureus* were examined in vitro. RT-qPCR was used to detect the changes in genes related to biofilm formation, whereas network pharmacology was used to predict the key targets and pathways of a cell-free supernatant of *B. velezensis* EA73 against *S. aureus*-mediated diseases. The minimum biofilm eradication concentration (MBEC) of the EA73 CFS against *S. aureus* was 1.28 × 10^−3^ g/mL. In addition, we observed that the EA73 CFS reduced bacterial adhesion and decreased extracellular proteins, polysaccharides, and the eDNA content in the biofilm and decreased the expression of biofilm-associated genes, such as *icaA* and *sarA*. The EA73 CFS had a significant effect on *S. aureus* biofilm eradication and hence can serve as a promising antibacterial reagent.

## 1. Introduction

*Staphylococcus aureus* is an important public health pathogenic bacterium and is the most commonly encountered bacterial species in infections [1]. The pathogenesis of this microorganism mainly involved virulence factors and biofilm formation. However, biofilms provide protection against host immune mechanisms and other antimicrobial agents during the adhesion and infection period, which makes it much more difficult to treat infections [2]. *S. aureus* biofilms are organized, surface-associated bacterial colonies enclosed in a self-secreting extracellular polymeric substance (EPS) matrix that adheres to biotic or abiotic surfaces. The EPS matrix consists primarily of extracellular polysaccharides, proteins, and eDNA [3]. Extracellular polysaccharides play a key role in the formation and stabilization of biofilms, and the polysaccharide intercellular adhesin (PIA) of *S. aureus* is an important extracellular polysaccharide [4]. PIA enables bacterial cells to adhere to each other agglomeratively, and it is an important substance in the early stage of biofilm formation for constructing the basic architecture, as it makes the mature biofilm have a three-dimensional structure [5]. *S. aureus* adhesion is also associated with biofilm formation and regulation of quorum sensing (QS) systems. The *agr* gene is a key component of the QS system of *S. aureus*. The autoinducing peptide (AIP) signaling molecules encoded by the *agr* gene and their signaling pathways constitute an important part of the QS system [6]. The number of AIP signaling molecules is positively correlated with the bacterial concentration, and when the bacterial concentration reaches a certain threshold, the QS system is activated [7]. Activation of this system can directly or indirectly regulate biofilm production and degradation, bacterial toxin secretion, and bacterial growth [8,9]. As the bacterial concentration rises, the QS system activates, prompting the generation of biofilms while activating the expression of virulence factors, such as the secretion of hemolytic toxins and proteases. These virulence factors can directly damage cells and trigger an inflammatory response, leading to inflammatory symptoms, such as redness, swelling, and pain [10]. Numerous studies have shown that the presence of *S. aureus* biofilm interferes with the normal wound repair process by occupying the wound site and preventing the growth of new cells and vascular tissue [11]. Additionally, bacteria embedded within biofilms can employ diverse mechanisms to elude the body’s immune clearance, further exacerbating the chronicity of inflammation. Hence, the presence of biofilms represents one of the crucial factors contributing to the persistence and poor resolution of bacterial infections.

Studies have shown that endophytes are common in all kinds of plants and are rich in species [12]. They produce many secondary metabolites with various biological activities, such as antibacterial and antitumor activities. They also improve plant vigor to diseases and insect pests [13,14]. Thus, endophytes can be a promising natural replacement for commercial antimicrobials. *Bacillus* spp. is one of the most important types of bacteria with inhibitory pathogen colonization, antimicrobial, immunomodulatory, and food fermentation capabilities [15]. Some species of *Bacillus* produce antimicrobial compounds, such as amino acids, enzymes, lipopeptides, bacteriocins, and bacteriocin-like compounds [16], and many of these antibacterial compounds are present in the CFS of Bacillus. In addition, some of the *Bacillus* spp. have the potential to be used as commercial antibiotic membrane reagents. For example, *Bacillus licheniformis* SPB-2 CFS and its derived silver nanoparticles have anti-biofilm activities against *S. aureus* and *Pseudomonas aeruginosa* [17]. Another study by Perini et al. [18] also indicated that *B. velezensis* 1273 CFS had antibacterial and anti-biofilm activities against *S. aureus* and methicillin-resistant *S. aureus*.

*B. velezensis* EA73, an endophyte from *Ageratina adenophora*, has shown excellent antibacterial activity against *S. aureus* [12]. However, the molecular mechanism involved in the endophytic bacteria’s antibacterial properties has not been elucidated yet. Therefore, in this study, we focused on investigating the molecular mechanisms associated with the anti-biofilm activity of *B. velezensis* EA73 CFS against *S. aureus* using in vitro methods and network pharmacology. This study will provide information on how *B. velezensis* EA73 CFS exhibits its antimicrobial activity to help us perform further studies to identify and harness secondary metabolites that are specific to the mechanism of action, thus preventing the issue of off targets and enhancing efficacy.

## 2. Materials and Methods

### 2.1. Strain and Growth Conditions

*B. velezensis* EA73 (GenBank no. MZ540895) was obtained from Hu Lab, Sichuan Agricultural University, and after routine passaging culture, it was preserved at −80 °C in a Luria–Bertani medium with 20% glycerol [12]. The *S. aureus* standard bacteria American Type Culture Collection (ATCC) 29213 was purchased as a lyophilized powder from Bioscibio (Hangzhou, China); activated; and preserved in tryptic soy broth (TSB) with 20% glycerol at −80 °C.

### 2.2. Preparation of EA73 CFS

The Ea73 activation solution was inoculated into a fermentation medium (yeast extract: 6.55 g/L; peptone: 6.61 g/L; NaCl: 20.00 g/L), and fermentation was carried out in a 180 r/min shaker according to fermentation parameters (initial pH: 7.95; temperature: 27.97 °C; time: 51.04 h) [12]. The fermentation broth was extracted several times with ethyl acetate at a ratio of 3:1, and the organic phase was collected and concentrated with a rotary evaporator at 45 °C under reduced pressure to obtain the concentrate, which was decontaminated with a 0.22 µm microporous membrane filter and then freeze-dried to obtain the dry substance (Freeze-dryer FDR2110, Rikaku eyela, Tokyo, Japan). The dry substance was stored at −20 °C until use.

### 2.3. In Vitro Experiment

#### 2.3.1. *S. aureus* Biofilm Formation Capacity

The ability of *S. aureus* to form biofilms was assessed via a crystal violet assay [18]. Bacteria were incubated on a constant temperature shaker for 8 h and then diluted with TSB. The OD_600_ values of the suspensions were measured using a spectrophotometer (Thermo NanoDrop One, Waltham, MA, USA), and the bacterial suspensions were diluted to the appropriate multiplicity for colony counting using the CFU counting method. Using an absorbance range of 0.1–1.0, the linear relationship between the absorbance (OD_600_) and the bacterial concentration was determined [19]. Two hundred microliters of sterile TSB and a 1% (*v*/*v*) *S. aureus* bacterial suspension (10^8^ CFU/mL; the bacterial suspension was diluted to an OD_600_ equal to 0.9) were added to 96-well plates and incubated at 37 °C for 4, 8, 12, 24, 48, 72, 96, or 120 h. After incubation, the medium was discarded, and the bacteria were washed with PBS to remove planktonic bacteria. Then, fixation was performed by adding 200 μL of methanol to each well for 1 h; the methanol was discarded, and the samples were washed with PBS. Then, 1% crystal violet was added to each well for 15 min and washed with PBS. The wells were dried on an ultraclean bench, and 200 μL of an ethanol–acetone mixture (4:1, *v*/*v*) was added. The absorbance was subsequently measured at 595 nm via a multifunctional fluorescence chemiluminescence immunometer (Thermo Scientific Varioskan Flash, Waltham, MA, USA). Wells with no bacteria were used as a blank group.

#### 2.3.2. Determination of MBEC

The MBEC of EA73 CFS against *S. aureus* was assessed using crystal violet staining and CFU counting [20]. A total of 200 µL of sterile TSB and a 1% (*v*/*v*) *S. aureus* bacterial suspension (10^8^ CFU/mL) were added to 96-well plates. The plates were incubated at 37 °C for 48 h to form a biofilm, and the supernatant was discarded. The plates were subsequently washed three times with PBS. A gradient concentration of EA73 CFS (0.16–10.24 × 10^−3^ g/mL) was added, and the mixture was incubated at 37 °C for 24 h. The culture medium was discarded, and the planktonic bacteria were rinsed with sterile PBS and discarded. Then, sterile PBS was added again, and the biofilm was suspended by repeated blowing. The suspension was diluted and spread evenly on a TSA plate for CFU counting. The results were observed after 24 h of incubation at 37 °C. The experiment was repeated three times, and the average value was taken.

After incubation of the biofilm and CFS treatment, the planktonic bacteria were rinsed with sterile PBS, and the biofilm was stained with 0.1% crystal violet for 15 min. After, it was rinsed with sterile PBS and dissolved with 95% ethanol. The OD value at 595 nm of the solution in the culture wells was determined via a multifunctional fluorescence chemiluminescence immunometer. MBEC has the lowest concentration of an antimicrobial substance that eradicates 99.9% of biofilm-embedded bacteria (3 log10 reduction in CFU/mL) compared to growth controls [21].

#### 2.3.3. Clearance of *S. aureus* Biofilms by EA73 CFS

The scavenging effect of EA73 CFS on *S. aureus* biofilms was assessed via crystal violet staining [22]. A total of 200 µL of sterile TSB and a 1% (*v*/*v*) *S. aureus* bacterial suspension (10^8^ CFU/mL) were added to 96-well plates. The plates were incubated at 37 °C for 48 h to form biofilms, after which the TSB was discarded, and then 200 µL of EA73 CFS at a concentration of 1/2MBEC, MBEC, 2MBEC was added, which corresponded to concentrations of 0.64 × 10^−3^ g/mL, 1.28 × 10^−3^ g/mL, and 2.56 × 10^−3^ g/mL, respectively. The wells to which 200 µL of the sterile TSB culture solution was added were used as a negative control, and the plates were placed in a thermostatic incubator for 12 h at 37 °C. The medium was discarded at 0 h, 1 h, 2 h, 4 h, 8 h, and 12 h, and the well plates were washed with 200 µL of sterile PBS 3–5 times and air-dried. A total of 100 µL of a methanol solution was added, and the mixture was fixed at room temperature for 15 min. The methanol was then discarded, and the mixture was allowed to dry naturally. Then, 100 µL of a crystal violet solution was added for 5 min at room temperature. The excess dye was washed with sterile PBS, and the mixture was air-dried. Next, 100 μL of a 33% glacial acetic acid solution was added, and the mixture was incubated at 37 °C for 30 min in a thermostatic incubator. The OD value at 595 nm of the solution in the culture wells was determined via a multifunctional fluorescence chemiluminescence immunometer.

#### 2.3.4. The Effects of EA73 CFS on the Micromorphology of *S. aureus* Biofilms

Five milliliters of sterile TSB, a 1% (*v*/*v*) *S. aureus* bacterial suspension (1 × 10^8^ CFU/mL), and one piece of sterile coverslip (2 cm × 2 cm) were added to 6-well plates. The culture was incubated at 37 °C for 48 h to form biofilms, after which the TSB was discarded, the biofilms were washed with sterile PBS, and then, EA73 CFS was added at concentrations of MBEC, which corresponded to concentrations of 1.28 × 10^−3^ g/mL, with the wells without EA73 CFS added as a control group. The plates were incubated at 37 °C for 4 h, and after incubation, the coverslips were removed, and cells were stained using the LIVE/DEAD Bacterial Staining Kit (Beyotime Co., Ltd., Shanghai, China), according to the instructions, and were analyzed using a fluorescence microscope (Olympus DP75, Tokyo, Japan) [20].

#### 2.3.5. The Effects of EA73 CFS on the Intramembrane Metabolism of *S. aureus* Biofilms

Resazurin has a weak fluorescence intensity, penetrates bacterial cell membranes, enters bacteria, and interacts with oxidoreductase enzymes in the body to be degraded into the highly fluorescent intermediate resorufin [23]. The conversion of resazurin to resorufin is irreversible and is proportional to the number of bacteria involved in metabolism, so this method can be used to indicate the viability of bacteria [24]. *S. aureus* biofilms were cultured on sterile coverslips, as described previously. The coverslips were removed and sonicated in sterile water for 45 min to separate the biofilm from the sterile coverslip sheet. A 10% resazurin solution was added to the bacterial suspension and was shaken for 2 h at 37 °C in the dark. The supernatant was then centrifuged at 10,000 rpm for 10 min at 4 °C, and the absorbance of the solution was detected by a multifunctional fluorescence chemiluminescence immunometer at an excitation wavelength of 560 nm and an emission wavelength of 590 nm [25,26].

#### 2.3.6. Analysis of EPSs

The biofilms were cultured on 6-well plates, according to the method described previously, and after incubation, the medium was discarded and washed slowly with sterile PBS to discard floating bacteria. Four groups were set up for the experiment, of which 1 group was a blank control group, and the remaining 3 groups were the experimental groups, and 3 wells were set up for each group. A total of 5 mL of sterile PBS was added to the blank control group, and equal amounts of sodium periodate (10 μM), DNaseI (2 mg/mL), and proteinase K (100 μg/mL) diluted to a fixed concentration with sterile PBS were added to the test group to perform enzyme digestion experiments [25,27]. The 6-well plates were then incubated at 37 °C for 2 h, and after, the medium was discarded. The planktonic bacteria were rinsed slowly with sterile PBS, and the biofilm was stained with crystal violet. The PBS was washed and decolorized with ethanol for 30 min, and the OD value of the solution at 595 nm was measured with a multifunctional fluorescence chemiluminescence immunometer.

Culturing of the biofilms was performed according to the methods described previously, and EA73 CFS (1/2 MBEC, MBEC, or 2 MBEC) was added while the bacteria were being cultured. After the incubation was complete, the TSB was discarded, and the mixture was rinsed with PBS to remove free bacteria from the surface. The cleaned coverslips were sonicated in PBS for 1 h, after which the bacterial suspension was centrifuged at 10,000 rpm for 10 min as an EPS sample for backup. Extracellular proteins were measured via the phenol-sulfuric acid method [28], and extracellular proteins were measured by the Coomassie Brilliant Blue method using a protein content assay kit (Biosharp Co., Ltd., Beijing, China) [29]. The eDNA was measured with a Bacterial Genomic DNA Rapid Extraction Kit (Tiangen Biotechnology Co., Ltd., Beijing, China). The absorbances of the solutions at OD_490_, OD_595_, and OD_260_ were detected via a multifunctional fluorescence chemiluminescence immunometer to analyze the contents of extracellular polysaccharides, extracellular proteins, and eDNA, respectively.

#### 2.3.7. Detection of Biofilm-Related Genes of *S. aureus*

*S. aureus* was cultured for 8 h, after which the bacterial suspension was diluted to 1 × 10⁸ CFU/mL, and then, EA73 CFS was added to the final concentration of MBEC. The bacteria suspension without CFS was used as a control group, and the RNA was extracted via the Bacterial Total RNA Extraction Kit (Tiangen Co., Ltd., Beijing, China) after incubation for 8 h at 37 °C. The RNA was reverse transcribed into cDNA via an Evo M-MLV Reverse Transcription Premix Kit (Accurate Co., Ltd., Changsha, China), followed by RT-qPCR via PerfectStart^®^ Green qPCR SuperMix (TransGen Co., Ltd., Beijing, China). Ten target genes among the polysaccharide intercellular adhesion (PIA) genes (*icaR*, *icaA*, and *icaD*), population-sensing genes (*agrA* and *agrC*), global regulators (*sarA*), adhesion-related genes (*clfA* and *clfB*), enolysis enzymes (*eno*), and protein-anchoring-related genes (*srtA*) were selected; 16S rRNA was used as the internal reference gene; and the primers used are listed in Table 1. The reaction program was as follows: predenaturation at 94 °C for 30 s, denaturation at 94 °C for 5 s, annealing at 60 °C for 30 s, extension at 72 °C for 10 s, and 45 cycles.

#### 2.3.8. Statistical Analysis

Experiments were performed in triplicate. Statistical analyses were conducted using GraphPad Prism 8 (GraphPad Inc., La Jolla, CA, USA). The results are shown as the mean ± standard error. Significant differences in the mean values were estimated by one-way analysis of variance (ANOVA) and Student’s *t*-test.

### 2.4. In Silico Experiment

#### 2.4.1. Component Analysis of EA73 CFS

To screen for possible active compounds in EA73 CFS, the sample was filtered through a 0.22 μm membrane, and the filtrate was added to assay vials and used for the assay. A Thermo Vanquish (Thermo Fisher Scientific, Waltham, MA, USA) ultra-high performance liquid chromatography (UPLC) system was used with an ACQUITY UPLC^®^ HSS T3 (2.1 mm × 100 mm, 1.8 µm) (Waters, Milford, MA, USA) column at a flow rate of 0.3 mL/min and a column temperature of 40 °C. The mobile phase was 0.1% formic acid in acetonitrile and 0.1% formic acid in water for the positive ionization mode and acetonitrile and 5 mM of ammonium formate in water for the negative ionization mode with gradient elution. A Thermo Q Exactive Focus mass spectrometry detector (Thermo Fisher Scientific, Waltham, MA, USA) equipped with an electrospray ionization (ESI) source was used, and data were collected separately in positive and negative ion modes. The positive ion spray voltage was 3.50 kV, the negative ion spray voltage was −2.50 kV, the sheath gas was 40 arb, and the auxiliary gas was 10 arb. The capillary temperature was 325 °C, and the primary full scan was performed at a resolution of 70,000, with a primary ion scanning range of *m*/*z* 100~1000, and the secondary cleavage was carried out using an HCD with a collision energy of 30 eV, and the secondary resolution was 17,500. The first 3 ions of the acquired signal were fragmented, while dynamic exclusion was used to remove unnecessary MS/MS information.

#### 2.4.2. Target Prediction and Collection

For the components identified in the previous subsection, the compounds with a relative content greater than 1% were selected, the SMILE names of the main components were obtained through pubchem (https://pubchem.ncbi.nlm.nih.gov/, accessed on 20 December 2024), and they were imported into the SWISSTargetPrediction database (http://www.swissadme.ch/, accessed on 20 December 2024), and “homo sapiens” was selected as the species, and then, the targets with Probability > 0 were screened as the gene targets. In the DisGeNET database (https://www.disgenet.com/, accessed on 20 December 2024) and the GeneCards database (https://genecards.org, accessed on 20 December 2024), the keyword “*Staphylococcus aureus*” was entered, and the genes with a relevance score > 5.0 were selected in the GeneCards database. In the GeneCards database, genes with a score > 5.0 were selected, and the results obtained from the two databases were integrated to remove duplicates as disease gene targets [30].

#### 2.4.3. Component–Target–Disease Network Analysis

The intersection of EA73 CFS main active ingredient targets and *S. aureus*-induced disease targets was taken through the Venny 2.1.0 website (https://bioinfogp.cnb.csic.es/tools/venny/, accessed on 20 December 2024). A component–target–disease network graph was constructed by using the Cytoscape 3.9.1 software, and the node sizes were adjusted according to the size of the degree using the cytoNCA plug-in to predict the key targets of EA73 CFS against *S. aureus*-induced diseases.

#### 2.4.4. Protein–Protein Interaction Network Construction

Through the STRING (https://string-db.org/, accessed on 20 December 2024) platform, we imported the common targets of the component genes and disease genes, set the object as “*Staphylococcus aureus*”, and hid the free gene nodes with the highest confidence level of 0.900 to obtain the interaction relationship of protein–disease interactions.

#### 2.4.5. GO and KEGG Enrichment Analyses

The DAVID database (https://david.ncifcrf.gov/, accessed on 20 December 2024) was used for an enrichment analysis of drug–disease shared targets, with “official gene symbol” as the identifier, “*S. aureus*” as the species, and the top 20 *p*-values as the screening criteria. The top 20 *p*-values were selected as the screening criteria, and the GO and KEGG enrichment analyses were performed by microbial letter mapping (https://www.bioinformatics.com.cn/, accessed on 20 December 2024). GO enrichment analysis describes the molecular functions performed by a gene product, the cellular environment in which it resides, and the biological processes in which it is involved. KEGG enrichment analysis identifies the most important biochemical metabolic pathways and the signaling pathways involved in the gene product. The results were also visualized through the microbiology platform.

#### 2.4.6. Molecular Docking Computer Simulation Verification

The key active ingredients screened in the “Ingredient–Target–Drug” network were used as ligands, and the core targets screened in the protein–protein interaction (PPI) network were used as receptors, and the structural files of the compounds were retrieved through the pubchem website (https://pubchem.ncbi.nlm.nih.gov/, accessed on 20 December 2024) and converted into PDB files using the Open Babel 2.3.2 software. The SDF files were converted into PDB files using the Open Babel 2.3.2 software. The receptor proteins were retrieved from the PDB database to obtain the receptor proteins. The PYMOL 2.3.4 software was used to dehydrate and de-ligand the receptor protein, and the AutoDockTools 4.2 software was used to modify the receptor protein with hydrogenation and a balanced charge, and the receptor protein and ligand small molecules were converted into pdbqt format, respectively. Molecular docking of receptor proteins and ligand small molecules was performed using AutoDock Vina 1.1.2, and the docking results were analyzed using PLIP. The docking results were visualized using PyMOL 2.6.

## 3. Results

### 3.1. In Vitro Experiment Results

#### 3.1.1. Biofilm Formation Time

**Figure 1:**
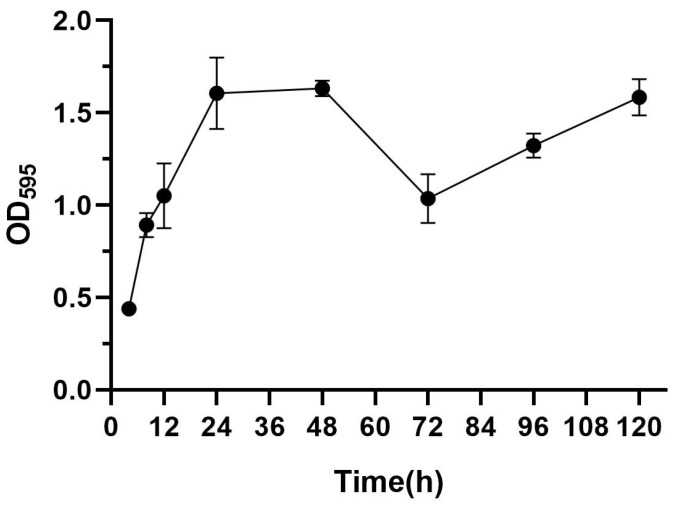
Biofilm formation ability of *S. aureus*.

Biofilm formation is divided into five stages (planktonic stage–reversible stage–irreversible stage–mature stage–dispersed stage) [31]. As shown in Figure 1, the OD values increased progressively with an increase in the incubation time, attaining a peak value of 1.63 at 48 h, indicative of a robust biofilm-forming capacity in this strain [32], and the OD peak suggests that the biofilm at this time reached the maturation stage. The OD value decreased at 72 h, and this stage corresponded to the dispersion stage of the biofilm, where part of the biofilm detached from the bacteria and returned to the planktonic state, dispersed to new parts of the bacteria, and carried out a new round of biofilm colonization at this point of disintegration and dispersal, which corresponded to the secondary elevation of the OD value at 92 h. Thus, in subsequent experiments, the biofilm incubation time was chosen to be 48 h.

#### 3.1.2. The Determination of the MBEC of *S. aureus* Biofilms by EA73 CFS

**Figure 2:**
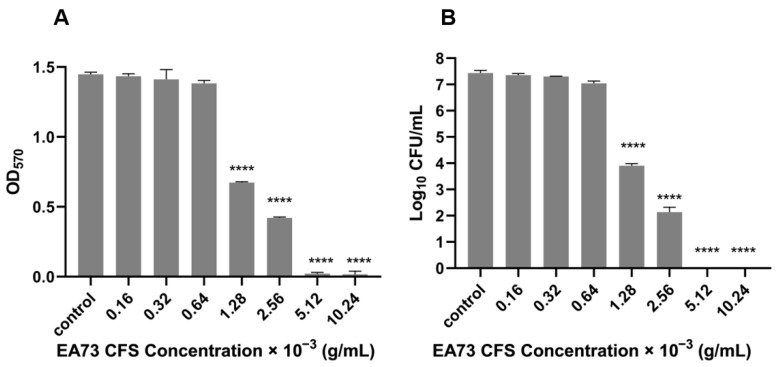
MBEC of EA73 CFS against *S. aureus* by crystal violet staining (**A**). MBEC of EA73 CFS against *S. aureus* by the CFU counting method (**B**). **** *p* < 0.0001 compared to control.

The MBEC of the EA73 CFS against *S. aureus* was determined via crystal violet staining and CFU counting. As shown in Figure 2, when the concentration of EA73 CFS reached 1.28 × 10^−3^ g/mL, the OD value of the experimental group was significantly lower than that of the control group (*p* < 0.0001), and the amount of biofilm was significantly reduced (Figure 2A). In the CFU counting method, at this concentration, the number of bacteria in the experimental group was reduced compared to the control group by 10^3.5^ CFU/mL (Figure 2B). The combined results show that the MBEC of EA73 CFS against *S. aureus* was 1.28 × 10^−3^ g/mL.

#### 3.1.3. Removal of *S. aureus* Biofilms by EA73 CFS

**Figure 3:**
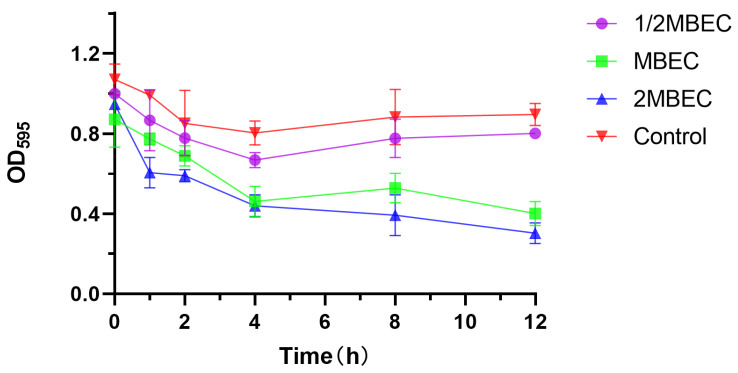
Elimination effect of EA73 CFS on the biofilm of *S. aureus* by the crystal violet semiquantitative method.

The experimental results are shown in Figure 3. The OD value of the blank group slightly decreased at 12 h, which might be related to the dispersion stage after biofilm maturation, and the OD_595_ value of the experimental group decreased with an increasing EA73 CFS concentration, indicating that the higher the concentration of EA73 CFS was, the greater the removal effect on *S. aureus* biofilms was. The biofilm removal rate reached 40.12% after 8 h of treatment at the MBEC concentration and 66.19% after 12 h of treatment at 2 MBEC, indicating that EA73 CFS is effective in removing *S. aureus* biofilms to a certain extent. The difference at point 0 may be related to insufficient mixing of the bacterial solution at the time of adding the samples or the loss of biofilm caused by the rinsing process of PBS.

#### 3.1.4. Effects of EA73 CFS on the Micromorphology of *S. aureus* Biofilms

**Figure 4:**
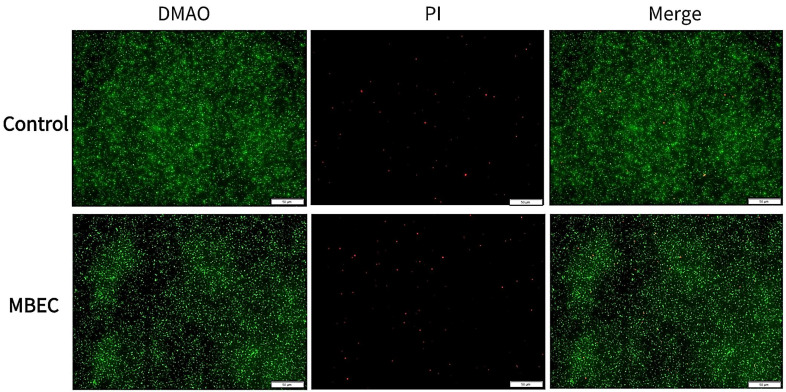
Representative *S. aureus* biofilms stained with LIVE/DEAD Kit and analyzed using fluorescence microscopy. The green fluorescence indicates the live cells, whereas the red fluorescence indicates the dead cells or cells with a damaged cell wall.

The fluorescence microscopy results are shown in Figure 4, where the untreated samples show the formation of mature biofilms characterized by aggregates of living cells with high fluorescence intensity. In contrast, *S. aureus* treated with an MBEC concentration of EA73 CFS was more dispersed with relatively lower fluorescence intensity.

#### 3.1.5. Effects of EA73 on Metabolism Within the Membrane of *S. aureus* Biofilms

**Figure 5:**
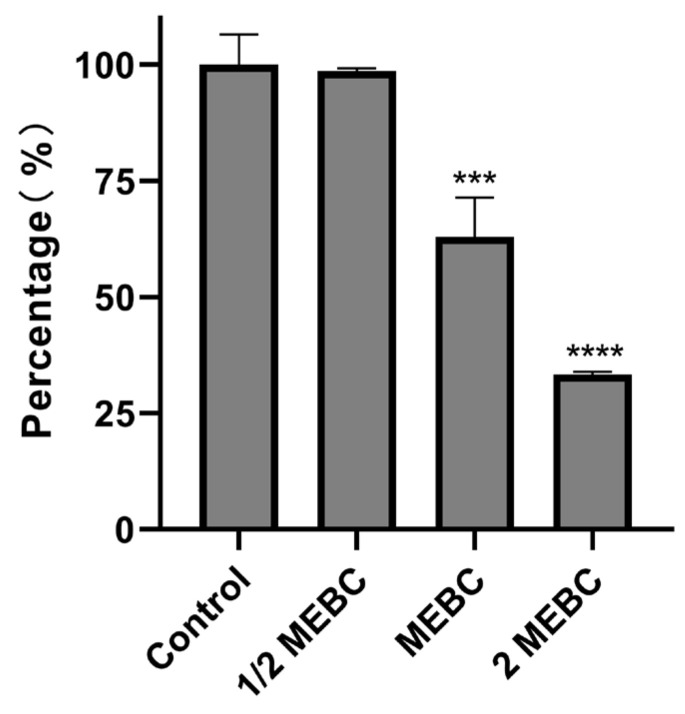
Effects of EA73 CFSs on the metabolism of *S. aureus* biofilms. *** *p* < 0.001 compared to control; **** *p* < 0.0001 compared to control.

As shown in Figure 5, the intramembrane metabolic activity of *S. aureus* biofilms decreased by 1%, 37%, and 67% after treatment with EA73 CFS at the concentrations of 1/2 MBEC, MBEC, and 2 MBEC, respectively.

#### 3.1.6. Effects of EA73 CFS on the Extracellular Polymers of *S. aureus* Biofilms

**Figure 6:**
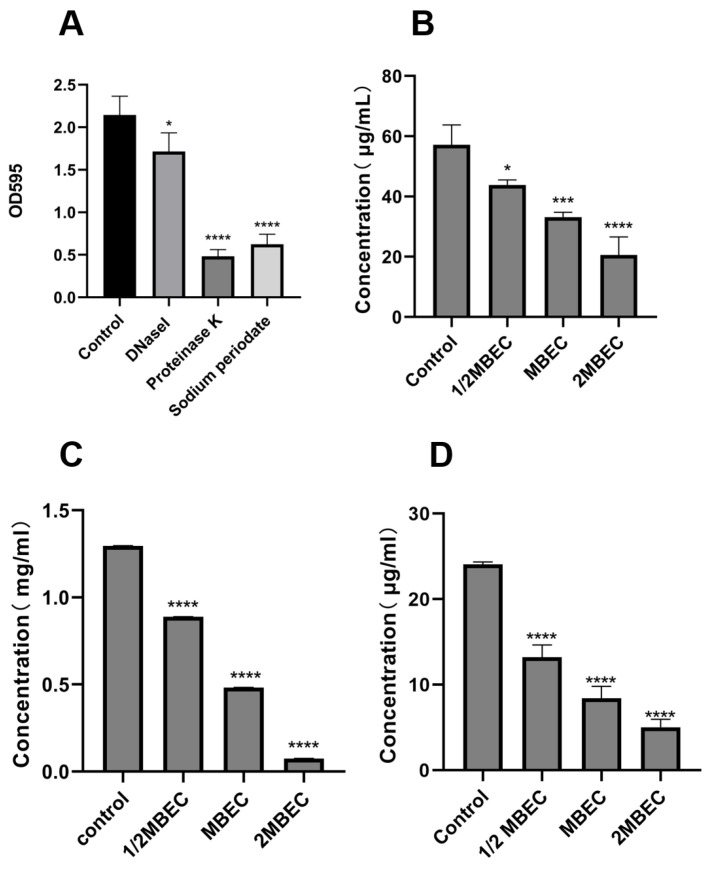
Enzymolysis experiment (**A**); effects of EA73 CFS on the extracellular polysaccharide (**B**), extracellular protein (**C**), and eDNA (**D**) content of *S. aureus* biofilms. * *p* < 0.05; *** *p* < 0.001; **** *p* < 0.000 compared to control.

EPS is a key factor in bacterial biofilm formation, and its composition varies with the species of microorganisms. Sodium periodate, DNase I, and Proteinase K hydrolyze polysaccharides, DNA, and proteins, respectively [33]. As shown in Figure 6A, enzymatic digestion experiments revealed that EA73 CFS reduced the levels of extracellular polymers of *S. aureus* biofilms with the order, from the highest to the lowest as proteinase, of K < sodium periodate < DNase I.

As shown in Figure 6B–D, the MBEC concentration of EA73 CFS reduced the concentrations of *S. aureus* extracellular polysaccharides, extracellular proteins, and eDNA by 62.81%, 41.94%, and 65.12%, respectively. In addition, the results show that EA73 CFS could cause changes in the composition of *S. aureus* EPS, which in turn altered the three-dimensional structure of the biofilm.

#### 3.1.7. Effects of EA73 CFS on the Expression of *S. aureus* Biofilm-Related Genes

**Figure 7:**
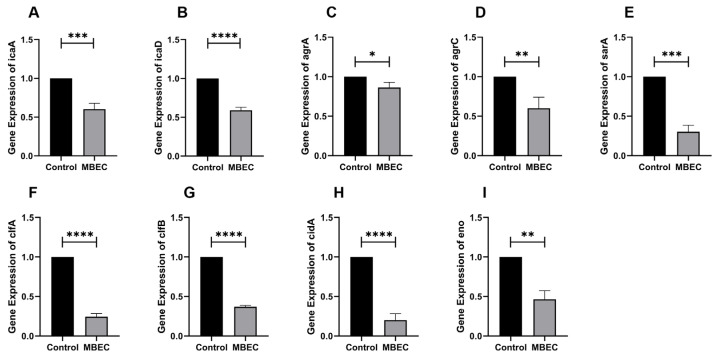
RT-qPCR results of *icaA* (**A**), *icaD* (**B**), *agrA* (**C**), agC (**D**), *sarA* (**E**), *clfA* (**F**), *clfB* (**G**), *cidA* (**H**), and *eno* (**I**) genes after EA73 CFS treatment. * *p* < 0.05; ** *p* < 0.01; *** *p* < 0.001; **** *p* < 0.0001 compared to control.

As shown in Figure 7, when 9 biofilm-related genes were transformed by MBEC EA73 CFS, the expression of *icaR* increased by 49.5%, and the expression of *icaA*, *icaD*, *agrA*, *agrC*, *sarA*, *cidA*, *clfA*, *clfB*, and *eno* decreased by 39.6%, 40.9%, 13.7%, 39.9%, 69.8%, 79.8%, 75.5%, 63.1%, and 53.7%, respectively.

### 3.2. In Silico Experiments

#### 3.2.1. LC-MS/MS Results

**Figure 8:**
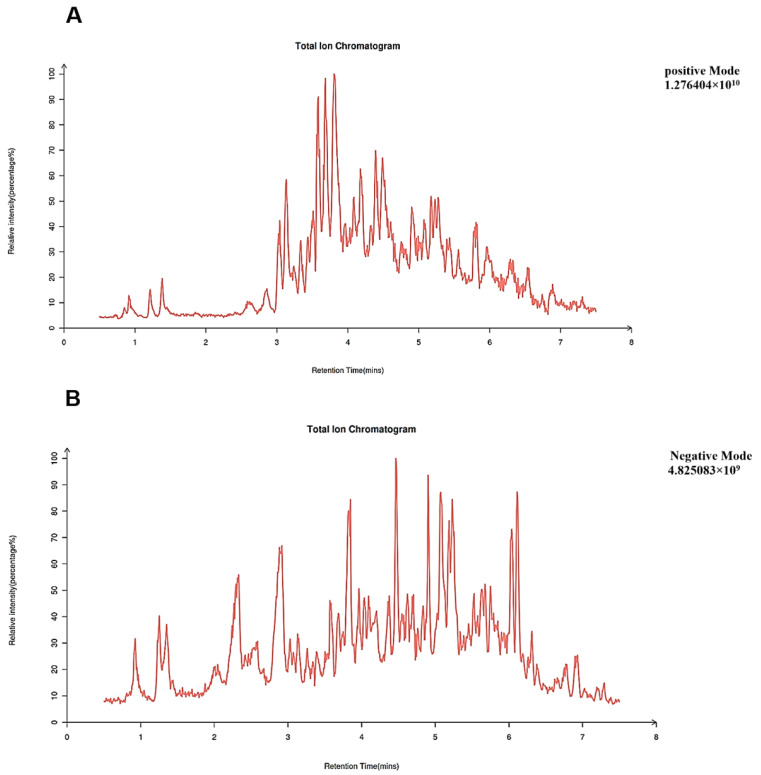
Total ion chromatogram, TIC. (**A**) Positive mode; (**B**) negative mode.

LC-MS/MS allowed for simultaneous qualitative and quantitative analysis of compounds in EA73 CFS biological samples. The peaks of 2004 metabolites were available for identification in EA73 CFS (Figure 8). As shown in Table 2, these peaks were mainly composed of phenolic compounds, aldehydes, organic acids, alkaloids, amino acids, flavonoids, and so on. Among them, 3,4-Dimethyl-2-(1-pyrrolidinyl)-2-cyclopenten-1-one (8.83%), Riboflavin (6.71%), 3,4-Dimethoxybenzaldehyde (5.49%), Ureidopropionic acid (5.12%), and Guanidoacetic acid (3.32%) had the highest content. There were 15 compounds with relative contents greater than 1%.

#### 3.2.2. Common Targets of EA73 CFS Main Ingredients and *S. aureus*-Mediated Diseases

**Figure 9:**
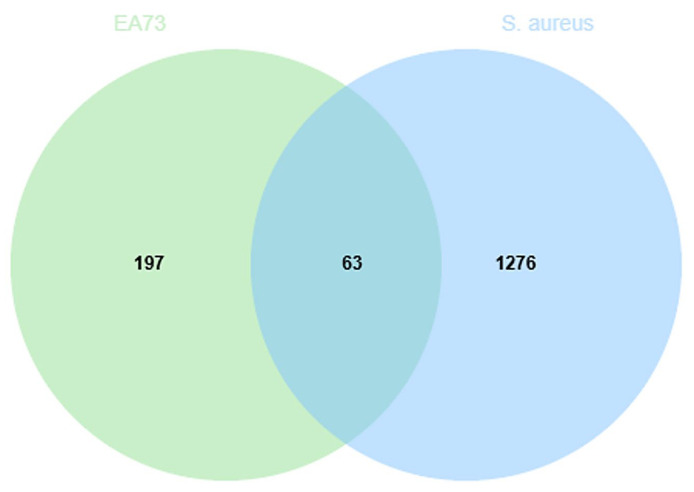
Venn diagram of intersection targets.

A Swiss TargetPrediction analysis (*p* > 0.5) identified 197 potential active ingredient targets of EA73 CFS and 1276 *S. aureus*-mediated disease targets through the GeneCards and DisGeNET databases and imported 197 EA73 CFS ingredient targets and 1276 *S. aureus*-mediated disease targets into Venny 2.1.0 to obtain 63 intersecting targets (Figure 9).

#### 3.2.3. Component–Target–Disease Network Diagram

**Figure 10:**
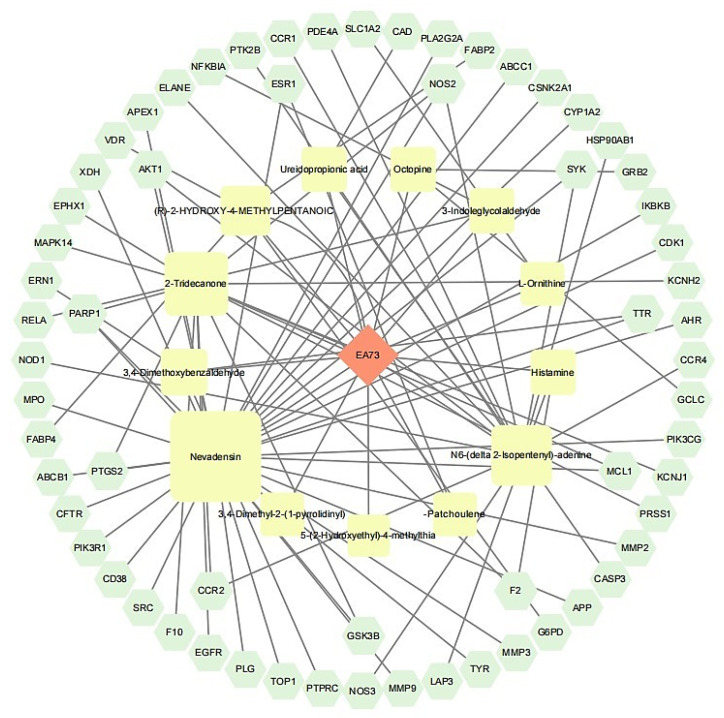
Network diagram of component–target–disease.

A total of 63 potential targets of the main components of the EA73 fermentation broth were obtained from the SwissTargetPrediction database. The main components and their corresponding targets were imported into Cytoscape 3.9.1 to draw the visualized network diagram (Figure 10), and the connected edges represent the correspondence of drug-active ingredient–target–disease interactions. Among them, Nevadensin, 2-Tridecanone, N6-(delta 2-Isopentenyl)-adenine, and (R)-2-Hydroxy-4-methylpentanoic acid had the most targets of action, with degree values of 33, 15, 14, and 5, respectively, which suggests that these components may play an important role in interactions with *S. aureus*-mediated diseases.

#### 3.2.4. PPI Network

**Figure 11:**
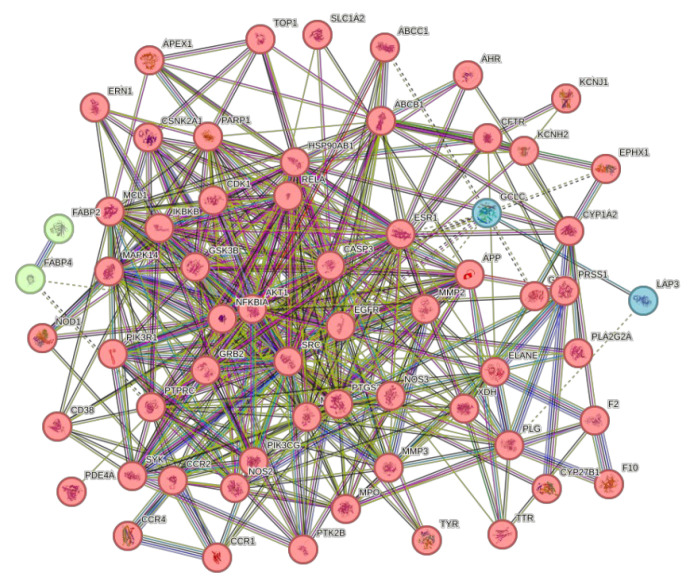
PPI network diagram.

The 63 intersecting targets were entered into the STRING database, and the species was selected as “*S. aureus*” by the minimum required interaction score >0.4, and the network display option was “hide disconnected nodes in the network”, and the K-mean clustering was selected as 3 to obtain its PPI network graph (Figure 11). This network graph has 63 nodes, 429 edges, and an average degree value of 13.6. The protein targets with higher degree values were AKT1, CASP3, EGFR, SRC, and MMP9, with degree values of 40, 35, 34, 34, and 33, respectively.

#### 3.2.5. Analysis of GO Function and KEGG Enrichment of Related Targets

**Figure 12:**
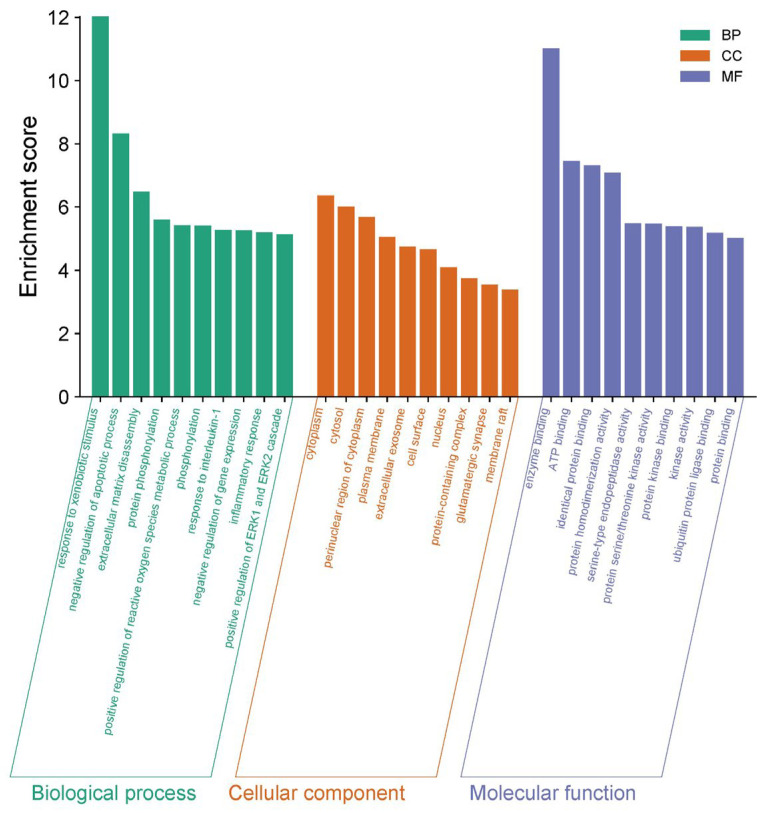
GO enrichment analysis of the corresponding targets.

Gene Ontology (GO) enrichment analysis yielded a total of 387 results, of which 272 were BP (Biological process), 43 were CC (Cellular component), and 72 were MF (Molecular function), which were screened according to −log10 (*p*-value) (Figure 12). Of these, BP was mainly associated with the response to exogenous stimuli, the negative regulation of apoptosis, extracellular matrix catabolism, protein phosphorylation, and the positive regulation of reactive oxygen species metabolism. CC was mainly associated with the cytoplasm, cytoplasmic lysate, cytoplasmic perinuclear region, and the cell membrane, and MF was associated with enzyme binding, ATP binding, homologous protein binding, protein homodimerization activity, etc.

**Figure 13:**
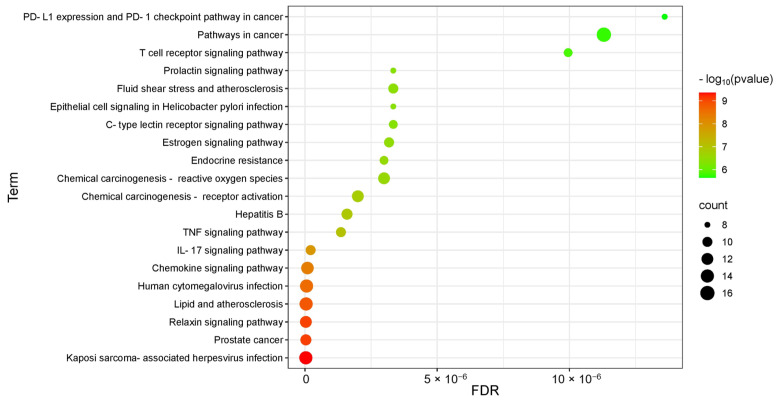
KEGG pathway analysis of the corresponding targets.

A total of 122 pathways were obtained from the Kyoto Encyclopedia of Genes and Genomes (KEGG) pathway enrichment analysis (Figure 13). The enrichment results indicate that the mechanism of action of EA73 CFS may be closely related to *Bordetella sarcoma*-associated herpesvirus infection, prostate cancer, the relaxin signaling pathway, lipids and atherosclerosis, human cytomegalovirus infection, the chemokine signaling pathway, the IL-17 signaling pathway, and other pathways.

#### 3.2.6. Molecular Docking Analysis

**Figure 14:**
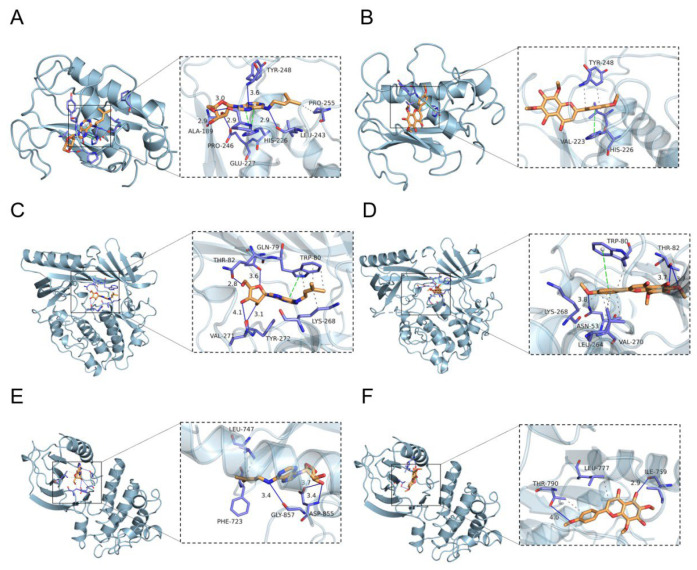
Partial diagram of molecular docking: (**A**) MMP9-N6-(delta2-Isopentenyl)-adenine; (**B**) MMP9-Nevadensin; (**C**) AKT1-N6-(delta2-Isopentenyl)-adenine; (**D**) AKT1-Nevadensin; (**E**) EGFRN6-(delta2-Isopentenyl)-adenine; (**F**) EGFR-Nevadensin.

The LC/MS-MS assay components and key targets were semi-flexibly docked by Autodock-vina, and the scores and interaction energy results obtained are shown in Table 3. In general, in the field of drug design, if the binding energy is lower than −7.0 kcal/mol, it is usually considered to have a relatively strong binding ability. The binding energies of the EA73 CFS active components and the key target proteins were both lower than −5.0 kcal/mol, indicating that the receptor binds the ligand well. Among them, MMP9, AKT1, and EGFRN6 showed very strong affinity to N6-(delta2-isopentenyl) and Nevadensin. These six pairs were selected for visualization in Figure 14, and hydrogen bonds were formed between all small molecules and receptor proteins, suggesting high structural stability upon binding.

## 4. Discussion

*S. aureus* is a commensal pathogen that is the most common pathogen isolated in cultures of skin-and-soft-tissue infections in the United States [34]. The presence of a biofilm leads to increased resistance of the bacteria to the external environment and can enhance bacterial adhesion to tissues and organs, leading to increased pathogenicity. Endophytic bacteria and their metabolites originating from natural plants can be used as an alternative to antibiotics. The focus of this study was to evaluate the effect of the endophytic bacterium EA73 CFS derived from *Ageratina adenophora* on *S. aureus* biofilms to provide new ideas for the prevention, control, and treatment of public health and safety issues related to *S. aureus* infections.

For the in vitro experiments, the biofilm-forming ability of the *S. aureus* ATCC 29213 strain isolated from human wounds was first determined. The results show that the OD value of the biofilm of this strain reached the highest level at the 48th hour of incubation, indicating that the biofilm was in the mature stage at this time. Bacteria at this stage are usually in the most difficult period to be treated due to the presence of physical barriers, changes in bacterial physiological characteristics, an increase in drug-resistant genes, and population sensing [35]. Therefore, the state of the biofilm after 48 h of incubation was chosen as the experimental condition for subsequent experiments. As shown in Figure 3, the inhibitory effect of the EA73 CFS on *S. aureus* biofilm was evident to a certain extent. In contrast to the extracellular substances of the *S. aureus* biofilm, bacteria possess a complex and well-ordered metabolic system within their cell membranes. In addition, in the presence of EA73 CFS, the metabolism of bacteria within the *S. aureus* biofilm decreased in a concentration-dependent manner (Figure 5), which is also consistent with a study by Afroj et al. [36], which reported that *B. velezensis* AP183 inhibits *S. aureus* biofilm formation. EPS is a key component and an important building block of the three-dimensional structure of bacterial biofilms [37]. As can be seen in Figure 6, the extracellular polysaccharides, extracellular proteins, and eDNA of the *S. aureus* EPS matrix were reduced by EA73 CFS, and the main structure of the *S. aureus* EPS matrix was disrupted. This finding is consistent with results that report that some *Bacillus* CFSs have a disruptive effect on the three-dimensional structure of *S. aureus* biofilms [38,39]. In many staphylococci, PIA is an important component of extracellular polysaccharides in their biofilms, and the synthesis of PIA is mediated by the *ica* manipulator gene [40]. Meanwhile, *clfA* and *clfB* genes encoding *S. aureus* surface adhesin proteins are also related to extracellular proteins in EPS, which are located on the surface of bacterial cells and mediate adhesion of bacteria to host cells or extracellular matrix components, playing an important role in biofilm formation and infection in *S. aureus* [41,42]. eDNA in EPS is related to the *cidA* gene. The CidA protein encoded by the *cidA* gene induces cell lysis, releasing intracellular DNA, and the DNA released outside the cell is an important source of eDNA [43,44]. The *eno* gene plays a key role in the glycolytic pathway of *S. aureus*, catalyzing the conversion of 2-phosphoglyceric acid to phosphoenolpyruvic acid, which produces high-energy molecules, such as ATP [35], and provides energy for bacterial survival, growth, physiological activities, and biofilm formation within the biofilm [45]. The reduction in *eno* gene expression is consistent with the reduction in metabolism within the biofilm after treatment with EA73 CFS in Figure 5. The *agr* system also plays an important role in regulating the expression of virulence factors in *S. aureus*; thus, a reduction in *agr* gene expression leads to biofilm disintegration. When the expression of *agrA* and *agrC* genes is inhibited, the transcription level of RNAIII will also decrease, which in turn affects the expression of virulence genes, thus breaking the mechanism that helps bacteria spread and cause disease in the host [46]. *sarA* is a global transcriptional regulator in *S. aureus*, and the presence of *sarA* enhances the transcription of the *ica* manipulator and promotes the production of PIA, thereby promoting biofilm formation [47]. Moreover, the *sarA* gene binds to a specific region of *agr*, which activates the transcription of *agr* [48]. The *ica* manipulator and *agr* gene expression changes are also in line with the effects caused by the decrease in the expression of the *sarA* gene. As shown in Figure 7, the expression of the *icaA*, *icaB*, *clfA*, *clfB*, *cidA sarA*, *agr*, and *eno* genes was decreased, and the results of the decrease in these genes are also consistent with the results of a study that showed that *Lactobacillus fermentum* TCUESC01 could decrease the *icaA* and *icaR* expression of *S. aureus* [49].

Among the top 20 compounds in the relative content of the LC-MS/MS results, β-Patchoulene and L-Ornithine had been reported to have anti-*S. aureus* bacteria and its anti-biofilm properties [50,51,52]. Indole-3-glycol aldehyde [53] and L-Leucyl-L-proline lactam [12] are currently being used as an antimicrobial drug by people in Iraq. Bergapten also has antibacterial bioactivity against *S. aureus* [54]. Riboflavin, as a kind of vitamin B group, helps to reduce oxidative stress and inflammation [55].

For the in-silico experiments in the GO analysis, the BP results suggest that the response to exogenous stimuli was associated with host defense against *S. aureus* infection, and EA73 CFS might regulate the activation of immune cells, such as macrophages and neutrophils, and the release of inflammatory factors to enhance bacterial clearance. During inflammation, substances such as proteases released by inflammatory cells lead to an extracellular matrix breakdown to facilitate the migration of immune cells and the diffusion of inflammatory mediators. Therefore, the enrichment of extracellular matrix breakdown-related genes may be associated with tissue remodeling and the progression of an inflammatory response during inflammation. In CC, the cytoplasm, cytoplasmic lysates, and the perinuclear region of the cytoplasm, processes such as transduction of inflammatory signals and synthesis and release of cytokines are closely related to these cellular components in inflammation. In KEGG pathway analysis, chemokines play a key role in inflammation, as they are able to attract immune cells, such as leukocytes and monocytes, to migrate towards the site of inflammation, thereby initiating and maintaining the inflammatory response [56]. The enrichment of the chemokine signaling pathway directly suggests that the EA73 CFS antimicrobial mechanism may be related to the process of immune cell recruitment in inflammation. This result is consistent with a previous study that reported that several strains of *Lactobacillus* spp. stimulate the inflammatory response and activate human macrophages after infection with pathogenic bacteria, such as *S. aureus*, *S. typhimurium*, and *E. coli* [57].

Molecular docking revealed that N6-(delta2-Isopentenyl)-adenine and Nevadensin ligands all bind to the active centers (catalytic sites or ATP-binding sites) of MMP9, AKT1, and EGFR target proteins, exerting their inhibitory effects through competitive inhibition or conformational interference. MMP9 is involved in the degradation and remodeling of the extracellular matrix. In *S. aureus* biofilm infections, its abnormal expression may contribute to biofilm formation and stabilization [58]. At the same time, MMP9 also regulates the migration and activation of inflammatory cells and promotes the release of inflammatory mediators [59], which in turn maintains the continuation of inflammatory responses. For example, it was reported that during inflammation, the activity and expression levels of MMP9 were significantly elevated in lesional tissues, which are positively correlated with the severity of inflammation [60]. AKT1, as a core kinase in the PI3K/AKT/mTOR signaling pathway, regulates host cell survival, metabolism, and immune responses [61]. Its overactivation can lead to immunosuppression or chronic inflammation [62]. Enhanced ligand binding affinity to AKT1 could disrupt AKT-mediated immunosuppressive signals, improve the bactericidal functions of immune cells (e.g., macrophages and neutrophils), inhibit excessive host cell proliferation, and reduce the necrotic tissue and biofilm matrix available for bacterial utilization. EGFR regulates epithelial cell proliferation, differentiation, and inflammatory factor release. Its activation promotes *S. aureus* invasion into host cells and contributes to chronic infections [63]. Ligands can inhibit the EGFR signaling pathway, reduce the release of pro-inflammatory factors, such as IL-6 and TNF-α, disrupt conditions favoring bacterial biofilm formation in inflammatory environments, and enhance the epithelial barrier function to impede bacterial adhesion.

The presence of *S. aureus* biofilms not only causes various infections but also inflammation and increased bacterial resistance. Therefore, the prevention of biofilm-associated infections is crucial. It is now known that *B. velezensis* EA73 CFS, which was mined from the endophytic bacteria of *Ageratina adenophora*, has anti-biofilm activity, and it is hypothesized that EA73 CFS exhibits anti-biofilm activity against *S. aureus* by suppressing the production of biofilms, inhibiting bacterial adherence, reducing the expression of virulence factor genes, and initiating immune cell recruitment. This suggests that EA73 CFS has great potential to address biofilm-associated problems in future clinical applications and could be used as a novel therapy for the treatment of *S. aureus*-induced infections. In future studies, the plan is to identify and isolate anti-biofilm-specific secondary metabolites and confirm their activity on various pathogenic bacteria.

## 5. Conclusions

In this study, it was demonstrated that *Bacillus velezensis* EA73 CFS had scavenging activity on *S. aureus* biofilms by reducing biofilm production and adhesion as well as repressing the transcription of biofilm-related genes. After knowing its main components, the targets and pathways of action of *Bacillus velezensis* EA73 CFS on *S. aureus*-mediated infections were explored using network pharmacology, and it was found that the CFS from *Bacillus velezensis* EA73 inhibits the activity of *S. aureus* by modulating the suppression of biofilm formation and decreasing virulence factors. Therefore, bioactive compounds with anti-biofilm activities can be isolated from *Bacillus velezensis* EA73. These results provide a theoretical basis for the development of new anti-biofilm agents against *S. aureus*.

## Figures and Tables

**Table 1:** Primers used for RT-qPCR in this study.

Genes	Sequence (5′-3′)
*16sRNA F*	ACTCCTACGGGAGGCAGCAG
*16sRNA R*	ATTACCGCGGCTGCTGG
*icaA F*	CTGGCGCAGTCAATACTATTTCGGGTGTCT
*icaA R*	GACCTCCCAATGTTTCTGGAACCAACATCC
*ica* *D F*	CCAGACAGAGGGAATACC
*ica* *D R*	AAGACACAAGATATAGCGATAAG
*agrA F*	TGATAATCCTTATGAGGTGCTT
*agrA R*	CACTGTGACTCGTAACGAAAA
*agrC F*	CGAAATGCGCAAGTTCCGT
*agrC R*	GTAGGCCAGGCATGTCATCT
*sarA F*	CAAACAACCACAAGTTGTTAAAGC
*sarA R*	TGTTTGCTTCAGTGATTCGTTT
*srtA F*	GAACCAGTATATCCAGGACCAGCAAC
*srtA R*	TAGTTCGGACGGTCAATGAAAGTGTG
*cidA F*	AGCGTAATTTCGGAAGCAACATCCA
*cidA R*	CCCTTAGCCGGCAGTATTGTTGGTC
*clf A F*	GCTTCAGTGCTTGTAGGTA
*clf A R*	GCTATCAGATTGCGTAACAC
*clf B F*	ACATCAGTAATAGTAGGGG
*clf B F*	TTCGCACTGTTTGTGTTTGCAC
*eno F*	AAACTGCAGTAGGTGACGAA
*eno R*	TGTTTCAACAGCATCTTCAGTACCTT

**Table 2:** Main components of EA73 CFS.

ID	Name	Rt (s)	Formula	Relative Content
M197T188_2	3,4-Dimethyl-2-(1-pyrrolidinyl)-2-cyclopenten-1-one	188.1	C_11_H_17_NO	8.83%
M377T196	Riboflavin	196.4	C_17_H_20_N_4_O_6_	6.71%
M165T173_1	3,4-Dimethoxybenzaldehyde	172.8	C_9_H_10_O_3_	5.49%
M131T138_2	Ureidopropionic acid	138.2	C_4_H_8_N_2_O_3_	5.12%
M116T335	Guanidoacetic acid	335	C_3_H_7_N_3_O_2_	3.32%
M243T367_2	2-Tridecanone	367.5	C_13_H_26_O	2.57%
M230T72_2	Octopine	72.2	C_9_H_18_N_4_O_4_	2.06%
M174T231	Indole-3-glycol aldehyde	230.6	C_10_H_9_NO_2_	2.06%
M186T93	N6-(delta 2-Isopentenyl)-adenine	93.2	C_10_H_13_N_5_	2.02%
M131T121_1	L-Ornithine	120.6	C_5_H_12_N_2_O_2_	1.55%
M144T86	5-(2-Hydroxyethyl)-4-methylthiazole	86.3	C_6_H_9_NOS	1.54%
M362T260_2	Nevadensin	260	C_18_H_16_O_7_	1.38%
M131T75_2	(R)-2-Hydroxy-4-methylpentanoic acid	75.1	C_6_H_12_O_3_	1.34%
M227T248	beta-Patchoulene	248.4	C_15_H_24_	1.32%
M221T362_2	Histamine	362.5	C_5_H_9_N_3_	1.17%
M320T349_2	19-Hydroxyandrost-4-ene-3,17-dione	349.1	C_19_H_26_O_3_	0.96%
M134T225	4-Methyl-1H-benzotriazole	225.5	C_7_H_7_N_3_	0.92%
M211T259	L-Leucyl-L-proline lactam	258.6	C_11_H_18_N_2_O_2_	0.85%
M247T304	Demethylmaprotiline	303.6	C_19_H_21_N	0.85%
M217T189	Bergapten	189.4	C_12_H_8_O_4_	0.72%

**Table 3:** Molecular docking score.

Receptor_Name	Ligand_Name	Scores
4xct-MMP9	2-Tridecanone	−6.5
4xct-MMP9	N6-(delta2-Isopentenyl)-adenine	−9.8
4xct-MMP9	Nevadensin	−9.3
5ias-CASP3	2-Tridecanone	−4.1
5ias-CASP3	N6-(delta2-Isopentenyl)-adenine	−6.7
5ias-CASP3	Nevadensin	−6.1
7nh5-AKT1	2-Tridecanone	−5.7
7nh5-AKT1	N6-(delta2-Isopentenyl)-adenine	−8.1
7nh5-AKT1	Nevadensin	−8.7
8a27-EGFR	2-Tridecanone	−6.3
8a27-EGFR	N6-(delta2-Isopentenyl)-adenine	−8
8a27-EGFR	Nevadensin	−8.4
8jn8-SRC	2-Tridecanone	−4.7
8jn8-SRC	N6-(delta2-Isopentenyl)-adenine	−6.9
8jn8-SRC	Nevadensin	−6.9

## Data Availability

The original contributions presented in this study are included in the article. Further inquiries can be directed to the corresponding author.

## Abbreviations

The following abbreviations are used in this manuscript:

CFS	Cell-free supernatant
EPS	Extracellular polymeric substance
PIA	Polysaccharide intercellular adhesin
QS	Quorum sensing
AIPs	Autoinducing peptides
MBEC	Minimum biofilm eradication concentration
DAPI	4′,6-Diamidino-2-phenylindole
GO	Gene Ontology
KEGG	Kyoto Encyclopedia of Genes and Genomes
PPI	Protein–protein interaction
BP	Biological process
CC	Cellular component
MF	Molecular function

## References

[1] Percival S.L., Emanuel C., Cutting K.F., Williams D.W. (2012). Microbiology of the skin and the role of biofilms in infection. Int. Wound J..

[2] Shen L., Zhang J., Chen Y., Rao L., Wang X., Zhao H., Wang B., Xiao Y., Yu J., Xu Y. (2023). Small-Molecule Compound CY-158-11 Inhibits *Staphylococcus aureus* Biofilm Formation. Microbiol. Spectr..

[3] Atshan S.S., Shamsudin M.N., Sekawi Z., Lung L.T.T., Hamat R.A., Karunanidhi A., Ali A.M., Ghaznavi-Rad E., Ghasemzadeh-Moghaddam H., Seng J.S.C. (2012). Prevalence of Adhesion and Regulation of Biofilm-Related Genes in Different Clones of *Staphylococcus aureus*. J. Biomed. Biotechnol..

[4] Chung P.Y., Loh P.L.N., Neoh H.-M., Ramli R. (2023). Alpha-amyrin as an anti-biofilm agent against methicillin-resistant and vancomycin-intermediate. *Staphylococcus aureus*. Heliyon.

[5] Polst B.H., Anlanger C., Risse-Buhl U., Larras F., Hein T., Weitere M., Schmitt-Jansen M. (2018). Hydrodynamics Alter the Tolerance of Autotrophic Biofilm Communities Toward Herbicides. Front. Microbiol..

[6] West K.H.J., Gahan C.G., Kierski P.R., Calderon D.F., Zhao K., Czuprynski C.J., McAnulty J.F., Lynn D.M., Blackwell H.E. (2022). Sustained Release of a Synthetic Autoinducing Peptide Mimetic Blocks Bacterial Communication and Virulence In Vivo. Angew. Chem.-Int. Ed..

[7] Paulander W., Varming A.N., Bojer M.S., Friberg C., Bak K., Ingmer H. (2018). The agr quorum sensing system in *Staphylococcus aureus* cells mediates death of sub-population. BMC Res. Notes.

[8] Caceres M., Hidalgo W., Stashenko E., Torres R., Ortiz C. (2020). Essential Oils of Aromatic Plants with Antibacterial, Anti-Biofilm and Anti-Quorum Sensing Activities against Pathogenic Bacteria. Antibiotics.

[9] Liu J.-Y., Jia J.-J., Liu M., Duan H., Hu M.-L., Liu C., Xue R.-Y., Jin Z.-L., Zhang S.-S., Li G.-C. (2023). A novel indolylbenzoquinone compound HL-J6 suppresses biofilm formation and α-toxin secretion in methicillin-resistant *Staphylococcus aureus*. Int. J. Antimicrob. Agents.

[10] Tamai M., Yamazaki Y., Ito T., Nakagawa S., Nakamura Y. (2023). Pathogenic role of the staphylococcal accessory gene regulator quorum sensing system in atopic dermatitis. Front. Cell. Infect. Microbiol..

[11] Uberoi A., McCready-Vangi A., Grice E.A. (2024). The wound microbiota: Microbial mechanisms of impaired wound healing and infection. Nat. Rev. Microbiol..

[12] Ren Z., Xie L., Okyere S.K., Wen J., Ran Y., Nong X., Hu Y. (2022). Antibacterial Activity of Two Metabolites Isolated From Endophytic Bacteria *Bacillus velezensis* Ea73 in *Ageratina adenophora*. Front. Microbiol..

[13] Strobel G., Daisy B., Castillo U., Harper J. (2004). Natural products from endophytic microorganisms. J. Nat. Prod..

[14] Lee C., Li W., Bang S., Lee S.J., Kang N.Y., Kim S., Kim T.I., Go Y., Shim S.H. (2019). Secondary Metabolites of The Endophytic Fungus *Alternaria alternata* JS0515 Isolated from *Vitex rotundifolia* and Their Effects on Pyruvate Dehydrogenase Activity. Molecules.

[15] Bajagai Y.S., Alsemgeest J., Moore R.J., Van T.T.H., Stanley D. (2020). Phytogenic products, used as alternatives to antibiotic growth promoters, modify the intestinal microbiota derived from a range of production systems: An in vitro model. Appl. Microbiol. Biotechnol..

[16] Ostovan R., Pourmontaseri M., Hosseinzadeh S., Shekarforoush S.S. (2021). Interaction between the probiotic *Bacillus subtilis* and *Salmonella* Typhimurium in Caco-2 cell culture. Iran. J. Microbiol..

[17] Karley D., Shukla S.K., Rao T.S. (2024). Biosynthesis of silver nanoparticle using *Bacillus licheniformis* culture-supernatant for combating pathogenic biofilms. Microb. Pathog..

[18] Ommen P., Zobek N., Meyer R.L. (2017). Quantification of biofilm biomass by staining: Non-toxic safranin can replace the popular crystal violet. J. Microbiol. Methods.

[19] Haaber J., Cohn M.T., Petersen A., Ingmer H. (2016). Simple method for correct enumeration of *Staphylococcus aureus*. J. Microbiol. Methods.

[20] Pourhajibagher M., Alaeddini M., Etemad-Moghadam S., Rahimi Esboei B., Bahrami R., Miri Mousavi R.s., Bahador A. (2022). Quorum quenching of *Streptococcus mutans* via the nano-quercetin-based antimicrobial photodynamic therapy as a potential target for cariogenic biofilm. BMC Microbiol..

[21] Dall G.F., Tsang S.J., Gwynne P.J., MacKenzie S.P., Simpson A., Breusch S.J., Gallagher M.P. (2018). Unexpected synergistic and antagonistic antibiotic activity against *Staphylococcus* biofilms. J. Antimicrob. Chemother..

[22] Perini H.F., Pereira B.B., Sousa E.G., Matos B.S., Silva Prado L.C.D., Carvalho Azevedo V.A., Castro Soares S., Silva M.V.D. (2024). Inhibitory effect of *Bacillus velezensis* 1273 strain cell-free supernatant against developing and preformed biofilms of *Staphylococcus aureus* and MRSA. Microb. Pathog..

[23] Narayanan K.B., Park G.T., Han S.S. (2021). Biocompatible, antibacterial, polymeric hydrogels active against multidrug-resistant *Staphylococcus aureus* strains for food packaging applications. Food Control.

[24] Bishop B.M., Juba M.L., Devine M.C., Barksdale S.M., Rodriguez C.A., Chung M.C., Russo P.S., Vliet K.A., Schnur J.M., van Hoek M.L. (2015). Bioprospecting the American Alligator (*Alligator mississippiensis*) Host Defense Peptidome. PLoS ONE.

[25] Li H., Li C., Ye Y., Cui H., Lin L. (2022). Inhibition mechanism of cyclo (L-Phe-L-Pro) on early stage *Staphylococcus aureus* biofilm and its application on food contact surface. Food Biosci..

[26] Jiang L.-M., Hoogenkamp M.A., van der Sluis L.W.M., Wesselink P.R., Crielaard W., Deng D.M. (2011). Resazurin Metabolism Assay for Root Canal Disinfectant Evaluation on Dual-species Biofilms. J. Endod..

[27] Cui H., Li H., Abdel-Samie M.A., Surendhiran D., Lin L. (2021). Anti-*Listeria monocytogenes* biofilm mechanism of cold nitrogen plasma. Innov. Food Sci. Emerg. Technol..

[28] Liu M., Wu X., Li J., Liu L., Zhang R., Shao D., Du X. (2017). The specific anti-biofilm effect of gallic acid on *Staphylococcus aureus* by regulating the expression of the *ica* operon. Food Control.

[29] Grintzalis K., Georgiou C.D., Schneider Y.-J. (2015). An accurate and sensitive Coomassie Brilliant Blue G-250-based assay for protein determination. Anal. Biochem..

[30] Liao F., Yousif M., Huang R., Qiao Y., Hu Y. (2023). Network pharmacology- and molecular docking-based analyses of the antihypertensive mechanism of *Ilex kudingcha*. Front. Endocrinol..

[31] Hong Q., Huo S., Tang H., Qu X., Yue B. (2021). Smart Nanomaterials for Treatment of Biofilm in Orthopedic Implants. Front. Bioeng. Biotechnol..

[32] Khasawneh A.I., Himsawi N., Abu-Raideh J., Salameh M.A., Al-Tamimi M., Al Haj Mahmoud S., Saleh T. (2020). Status of Biofilm-Forming Genes among Jordanian Nasal Carriers of Methicillin-Sensitive and Methicillin-Resistant *Staphylococcus aureus*. Iran. Biomed. J..

[33] Xue X., Sztajer H., Buddruhs N., Petersen J., Rohde M., Talay S.R., Wagner-Döbler I. (2011). Lack of the delta subunit of RNA polymerase increases virulence related traits of *Streptococcus* mutans. PLoS ONE.

[34] Ray G.T., Suaya J.A., Baxter R. (2013). Incidence, microbiology, and patient characteristics of skin and soft-tissue infections in a U.S. population: A retrospective population-based study. BMC Infect. Dis..

[35] Peng Q., Tang X., Dong W., Sun N., Yuan W. (2022). A Review of Biofilm Formation of *Staphylococcus aureus* and Its Regulation Mechanism. Antibiotics.

[36] Afroj S., Brannen A.D., Nasrin S., Al Mouslem A., Hathcock T., Maxwell H., Rasmussen-Ivey C.R., Sandage M.J., Davis E.W., Panizzi P. (2021). *Bacillus velezensis* AP183 Inhibits *Staphylococcus aureus* Biofilm Formation and Proliferation in Murine and Bovine Disease Models. Front. Microbiol..

[37] Kranjec C., Morales Angeles D., Torrissen Marli M., Fernandez L., Garcia P., Kjos M., Diep D.B. (2021). *Staphylococcal* Biofilms: Challenges and Novel Therapeutic Perspectives. Antibiotics.

[38] Zhang F., Wang B., Liu S., Chen Y., Lin Y., Liu Z., Zhang X., Yu B. (2021). *Bacillus subtilis* revives conventional antibiotics against *Staphylococcus aureus* osteomyelitis. Microb. Cell Factories.

[39] Park Y.J., Kim Y.J., Yu H.H., Lee N.-K., Paik H.-D. (2023). Cell-free supernatants of *Bacillus subtilis* and *Bacillus polyfermenticus* inhibit *Listeria monocytogenes* biofilm formation. Food Control.

[40] Nguyen H.T.T., Nguyen T.H., Otto M. (2020). The *staphylococcal* exopolysaccharide PIA–Biosynthesis and role in biofilm formation, colonization, and infection. Comput. Struct. Biotechnol. J..

[41] Hamushan M., Yu J., Jiang F., Wang B., Li M., Hu Y., Wang J., Wu Q., Tang J., Han P. (2024). Adaptive evolution of the Clf-Sdr subfamily contributes to *Staphylococcus aureus* musculoskeletal infection: Evidence from comparative genomics. Microbiol. Res..

[42] Preda M., Mihai M.M., Popa L.I., Ditu L.-M., Holban A.M., Manolescu L.S.C., Popa G.-L., Muntean A.-A., Gheorghe I., Chifiriuc C.M. (2021). Phenotypic and genotypic virulence features of *staphylococcal* strains isolated from difficult-to-treat skin and soft tissue infections. PLoS ONE.

[43] Qi M., Liu Q., Liu Y., Yan H., Zhang Y., Yuan Y. (2022). *Staphylococcus aureus* biofilm inhibition by high voltage prick electrostatic field (HVPEF) and the mechanism investigation. Int. J. Food Microbiol..

[44] Kolodkin-Gal I., Verdiger R., Shlosberg-Fedida A., Engelberg-Kulka H. (2009). Differential Effect of *E. coli* Toxin-Antitoxin Systems on Cell Death in Liquid Media and Biofilm Formation. PLoS ONE.

[45] Kot B., Sytykiewicz H., Sprawka I. (2018). Expression of the Biofilm-Associated Genes in Methicillin-Resistant *Staphylococcus aureus* in Biofilm and Planktonic Conditions. Int. J. Mol. Sci..

[46] Shompole S., Henon K.T., Liou L.E., Dziewanowska K., Bohach G.A., Bayles K.W. (2003). Biphasic intracellular expression of *Staphylococcus aureus* virulence factors and evidence for *Agr*-mediated diffusion sensing. Mol. Microbiol..

[47] Sang H., Jin H., Song P., Xu W., Wang F. (2024). Gallic acid exerts antibiofilm activity by inhibiting methicillin-resistant *Staphylococcus aureus* adhesion. Sci. Rep..

[48] Jiang M., Li Y., Sun B., Xu S., Pan T., Li Y. (2023). Phage transcription activator RinA regulates *Staphylococcus aureus* virulence by governing *sarA* expression. Genes Genom..

[49] Melo T.A., Dos Santos T.F., de Almeida M.E., Junior L.A., Andrade E.F., Rezende R.P., Marques L.M., Romano C.C. (2016). Inhibition of *Staphylococcus aureus* biofilm by *Lactobacillus* isolated from fine cocoa. BMC Microbiol..

[50] Junren C., Xiaofang X., Mengting L., Qiuyun X., Gangmin L., Huiqiong Z., Guanru C., Xin X., Yanpeng Y., Fu P. (2021). Pharmacological activities and mechanisms of action of *Pogostemon cablin Benth*: A review. Chin. Med..

[51] Mir M.A., Altuhami S.A., Mondal S., Bashir N., Dera A.A., Alfhili M.A. (2023). Antibacterial and Antibiofilm Activities of β-Lapachone by Modulating the Catalase Enzyme. Antibiotics.

[52] Pan M., Lu C., Zheng M., Zhou W., Song F., Chen W., Yao F., Liu D., Cai J. (2020). Unnatural Amino-Acid-Based Star-Shaped Poly(l-Ornithine)s as Emerging Long-Term and Biofilm-Disrupting Antimicrobial Peptides to Treat *Pseudomonas aeruginosa*-Infected Burn Wounds. Adv. Healthc. Mater..

[53] Gurkok G., Altanlar N., Suzen S. (2009). Investigation of Antimicrobial Activities of Indole-3-Aldehyde Hydrazide/Hydrazone Derivatives. Chemotherapy.

[54] Aldulaimi O. (2017). Screening of Fruits of Seven Plants Indicated for Medicinal Use in Iraq. Pharmacogn. Mag..

[55] Lee K., Choi Y.I., Im S.T., Hwang S.M., Lee H.K., Im J.Z., Kim Y.H., Jung S.J., Park C.K. (2021). Riboflavin Inhibits Histamine-Dependent Itch by Modulating Transient Receptor Potential Vanilloid 1 (TRPV1). Front. Mol. Neurosci..

[56] Smith J.S., Rajagopal S., Atwater A.R. (2018). Chemokine Signaling in Allergic Contact Dermatitis: Toward Targeted Therapies. Dermatitis.

[57] Rocha-Ramírez L.M., Pérez-Solano R.A., Castañón-Alonso S.L., Moreno Guerrero S.S., Ramírez Pacheco A., García Garibay M., Eslava C. (2017). Probiotic *Lactobacillus* Strains Stimulate the Inflammatory Response and Activate Human Macrophages. J. Immunol. Res..

[58] Julovi S.M., McKelvey K., Minhas N., Chan Y.-K.A., Xue M., Jackson C.J. (2024). Involvement of PAR-2 in the Induction of Cell-Specific Matrix Metalloproteinase-2 by Activated Protein C in Cutaneous Wound Healing. Int. J. Mol. Sci..

[59] Gong Y., Hart E., Shchurin A., Hoover-Plow J. (2008). Inflammatory macrophage migration requires MMP-9 activation by plasminogen in mice. J. Clin. Investig..

[60] Michalak-Stoma A., Bartosińska J., Raczkiewicz D., Kowal M., Krasowska D., Chodorowska G. (2021). Assessment of Selected Matrix Metalloproteinases (MMPs) and Correlation with Cytokines in Psoriatic Patients. Mediat. Inflamm..

[61] Bancroft T., Bouaouina M., Roberts S., Lee M., Calderwood D.A., Schwartz M., Simons M., Sessa W.C., Kyriakides T.R. (2015). Up-regulation of Thrombospondin-2 in Akt1-null Mice Contributes to Compromised Tissue Repair Due to Abnormalities in Fibroblast Function. J. Biol. Chem..

[62] Han H.-M., Kim S.-J., Kim J.-S., Kim B.H., Lee H.W., Lee Y.T., Kang K.-H. (2016). Ameliorative effects of *Artemisia argyi* Folium extract on 2,4-dinitrochlorobenzene-induced atopic dermatitis-like lesions in BALB/c mice. Mol. Med. Rep..

[63] An Z., Aksoy O., Zheng T., Fan Q.W., Weiss W.A. (2018). Epidermal growth factor receptor and EGFRvIII in glioblastoma: Signaling pathways and targeted therapies. Oncogene.

